# P-626. Relative Vaccine Effectiveness of Cell-Based Versus Egg-Based Quadrivalent Influenza Vaccines Against Test-Confirmed Influenza in the United States 2022-23 Influenza Season

**DOI:** 10.1093/ofid/ofae631.824

**Published:** 2025-01-29

**Authors:** Alicia N Stein, Anusorn Thanataveerat, Kimberly McDermott, Alex Dean, Stephanie Wall, Cory Pack, Ian McGovern, Sheena G Sullivan, Mendel Haag

**Affiliations:** CSL Seqirus, Melbourne, Victoria, Australia; Veradigm, New York, New York; Veradigm, New York, New York; Veradigm, New York, New York; Veradigm, New York, New York; Veradigm, New York, New York; CSL Seqirus, Melbourne, Victoria, Australia; University of California, Los Angeles, Melbourne, Victoria, Australia; CSL Seqirus, Melbourne, Victoria, Australia

## Abstract

**Background:**

Egg-based influenza vaccine viruses often acquire egg-adaptive mutations in the hemagglutinin protein that can alter their antigenicity and may contribute to reduced vaccine effectiveness. Cell-based vaccine manufacturing avoids egg adaptation. We recently demonstrated improved effectiveness of cell-based quadrivalent influenza vaccines (QIVc) compared with egg-based quadrivalent influenza vaccines (QIVe) in preventing test-confirmed influenza during the 2017-18 to 2019-20 influenza seasons in the population aged 4-64 years in the United States. The objective of this study was to estimate the relative vaccine effectiveness (rVE) of QIVc versus QIVe in preventing test-confirmed influenza in an extended population aged 6 months to 64 years during the 2022-23 season in the United States.

**Methods:**

The study applied a retrospective test-negative design among individuals aged 6 months to 64 years vaccinated with either QIVc or QIVe in 2022-23 and who had an influenza test obtained in routine outpatient care within +/- 7 days of a documented acute respiratory or febrile illness. Exposure, outcome, and covariate data were obtained from outpatient electronic health records linked to pharmacy and medical claims. rVE was estimated by comparing the odds of testing positive for influenza among QIVc recipients with the odds among QIVe recipients, using a doubly robust analysis. Sensitivity analyses included additional adjustment for the propensity to be tested, limiting to the peak influenza epidemic period, and matching on the test-week.

**Results:**

The study included 43,086 tested patients, of whom 18.6% received QIVc and 81.4% received QIVe. QIVc was more effective than QIVe in preventing test-confirmed influenza in the outpatient care setting, with an estimated rVE of 7.7% (95% CI: 0.9% – 13.9%). Preliminary results of sensitivity analyses are consistent with the main analysis.

**Conclusion:**

This study demonstrated superior effectiveness of QIVc compared to QIVe in preventing outpatient test-confirmed influenza in the population aged 6 months to 64 years during the 2022-23 season in the United States. These findings add to the body of evidence supporting improved effectiveness of cell-based versus egg-based influenza vaccines.

**Disclosures:**

**Alicia N. Stein, MBiostats, PhD**, CSL Seqirus: Employee|CSL Seqirus: Stocks/Bonds (Public Company) **Anusorn Thanataveerat, DrPH**, Veradigm: Employee of Veradigm, which received a research contract to conduct this study with and on behalf of CSL Seqirus **Kimberly McDermott, PhD**, Veradigm: Employee of Veradigm, which received a research contract to conduct this study with and on behalf of CSL Seqirus **Alex Dean, MPH**, Veradigm: Employee of Veradigm, which received a research contract to conduct this study with and on behalf of CSL Seqirus **Stephanie Wall, MPH**, Veradigm: Employee of Veradigm, which received a research contract to conduct this study with and on behalf of CSL Seqirus **Cory Pack, BS**, Veradigm: Employee of Veradigm, which received a research contract to conduct this study with and on behalf of CSL Seqirus **Ian McGovern, MPH**, CSL Seqirus: Employee|CSL Seqirus: Stocks/Bonds (Public Company) **Sheena G. Sullivan, PhD**, CSL: Advisor/Consultant|Moderna: Advisor/Consultant|Novavax: Advisor/Consultant|Pfizer: Advisor/Consultant **Mendel Haag, PhD, PharmD**, CSL Seqirus: Employment|CSL Seqirus: Stocks/Bonds (Public Company)

